# Spontaneous recurrence of a summer-like diel rhythm in the body temperature of the Syrian hamster after hibernation

**DOI:** 10.1098/rspb.2023.0922

**Published:** 2023-10-18

**Authors:** Satoshi Nakagawa, Yoshifumi Yamaguchi

**Affiliations:** ^1^ Graduate School of Environmental Sciences, Hokkaido University, Sapporo 060-0819, Japan; ^2^ Institute of Low Temperature Science, Hokkaido University, Sapporo 060-0819, Japan; ^3^ Inamori Research Institute for Science, Kyoto 600-8411, Japan

**Keywords:** hibernation, mammal, seasonal adaptation, body temperature, diel rhythm, Syrian hamster

## Abstract

Mammalian hibernation is a survival strategy characterized by metabolic suppression and drastically lowering body temperature (Tb), used during harsh seasons with food shortages and cold. The Syrian hamster commences hibernation in response to a short photoperiod and cold but spontaneously concludes hibernation after several months without environmental cues. Little is known about the changes in diel rhythms during hibernation. Using long-term and high-resolution Tb data, we analysed the diel Tb rhythm time-course changes in Syrian hamsters raised under summer-like conditions (long photoperiod (LP) and warm; LP-warm) and transferred to winter-like conditions (short photoperiod (SP) and cold; SP-cold). The diel Tb rhythm was undetectable during the hibernation period (HIBP), reappearing after the HIBP. The phase of this returning rhythm reverted to the LP entrainment phase characteristics despite the ambient SP and then re-entrained to the ambient SP as if the hamsters were transferred from the LP-warm to SP-cold conditions. The diel Tb rhythm reverted from the SP- to LP-type in a hibernation-dependent manner. Under constant dark and cold conditions, the circadian Tb rhythm recovered without photic stimuli following the HIBP. These findings suggest that hibernation involves a program that anticipates the ambient photoperiod when animals emerge from hibernation.

## 1. Introduction

**Figure 1.**
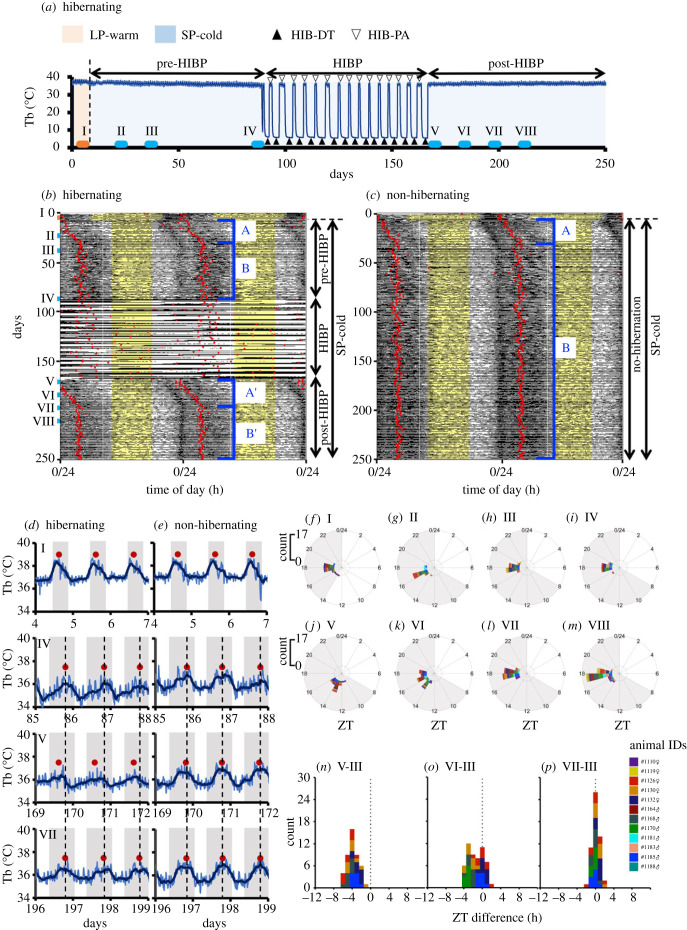
Hibernation-dependent cancellation of entrainment to the short photoperiod (SP) and subsequent re-entrainment of the diel body temperature (Tb) rhythm to the SP. (*a*) Time-course changes of the Tb in a hibernating individual (#1168♂). The deep blue line indicates the Tb value smoothed by a 4 h moving average filter. The periods I–VIII are shown and analysed in Figures *d*, *f–p*. Periods: (I) LP-warm condition; (II) 2 weeks and (III) 4 weeks after transfer from the LP-warm to SP-cold conditions; (IV) immediately before the onset of the HIBP; (V) immediately after the end of the HIBP; (VI) 2 weeks, (VII) 4 weeks and (VIII) 6 weeks after the end of the HIBP. (*b,c*) Representative double-plot actograms of Tb using a 24 h time window. (*b*) Hibernating (#1168♂) or (*c*) non-hibernating individuals (#1167♂) under prolonged SP-cold are shown. The black portion in each line of the actograms indicates the value of the Tb, which is normalized to its maximum and minimum line value. Specifically, in each line of the actogram, the zero and maximum values of the Y-axis correspond to the minimum and maximum values of the Tb within the line. The yellow shaded area in the actogram indicates the light phase. The red dots indicate the acrophases of the diel Tb rhythm fitted by a sine function at 24 h of τ. (*d,e*) Tb transitions of #1168 (*d*) and #1167 (*e*) in the periods I, IV, V and VII. These two hamsters were simultaneously transferred from LP-warm to SP-cold conditions. Dashed lines in (*d*) and (*e*) indicate the time at which the acrophases were observed in periods IV and VII. The shaded areas indicate the dark phases. The closed square brackets A, B, A’, and B’ indicate the following periods: A) The period after the transition from LP-warm to SP-cold conditions, during which the acrophase of the diel rhythm is gradually entrained to the SP. B) The period after SP entrainment. The acrophases were fixed in the second half of the dark phase before the HIBP. A’) The period during which SP entrainment was cancelled, following hibernation refractoriness and the acrophases were re-entrained to the SP. B’) The period after the SP re-entrainment, during which the acrophase was fixed again in the second half of the dark phase after the HIBP. (*f*–*m*) Polar plots showing the distribution of the acrophases of the diel Tb rhythm during periods I–VIII. Time is shown by zeitgeber time (ZT). The shaded areas indicate the dark phases. Each animal is represented by a different colour. (*n*–*p*) The ZT difference in acrophases between periods III and V (*n*), VI (*o*) or VII (*p*). Each animal is represented by a different colour.

Mammals adapt their physiology to the seasonal changes. Warm seasons with abundant food, such as spring and summer in higher-latitude regions, are favoured for offspring growth [1]. By contrast, the cold seasons with food scarcity, namely winter, pose severe challenges for survival. In particular, endotherms, such as mammals and birds, generate heat from their food energy intake to maintain their core body temperature (Tb) at much higher levels than the ambient temperature. Mammalian hibernation is a survival strategy in which some mammals choose to survive harsh seasons through the drastic systemic suppression of metabolism and energy expenditure [2,3]. In small-bodied hibernators, including hamsters, ground squirrels, and chipmunks, hibernation consists of cycles of hypothermic deep torpor bouts (HIB-DT) and normothermic periodic arousal (HIB-PA) during the hibernation period (HIBP; figure 1*a*). An episode of HIB-DT, during which metabolism, heart rate, and locomotor activity are profoundly suppressed [2,3], lasts several days to weeks in a species- and ambient-temperature-dependent manner [4]. HIB-DT is interrupted by the rapid recovery of Tb to approximately 37°C due to spontaneous heat production, resulting in HIB-PA with restored metabolism, heart rate and locomotor activity. HIB-PA lasts for approximately 1 day [4], after which the animal enters HIB-DT again. The HIBP lasts for several months or longer. Mammalian hibernators conclude the HIBP with the arrival of spring and become active again in their natural environment. This exit from the HIBP can occur without environmental cues under constant laboratory conditions, suggesting that mammalian hibernators possess endogenous timekeeping systems that determine the timing of the HIBP exit.

The system by which hibernation length is determined is unknown; however, the most promising candidate for such a system is the internal annual rhythm. Some hibernators, including ground squirrels, chipmunks, alpine marmots and bears, exhibit a circannual (Type II) rhythm of hibernation; furthermore, they display gonadal development and body weight control on an approximate annual cycle when kept under constant conditions [5], and are therefore designated as obligate hibernators [6–11]. By contrast, Syrian hamsters exhibit Type I annual rhythms that are influenced by both environmental changes and endogenous mechanisms [5]. Specifically, Syrian hamsters initiate hibernation throughout the year after several months of exposure to chronic winter-like conditions [short photoperiod (SP) and cold temperatures in a laboratory] of the pre-hibernation period (pre-HIBP; figure 1*a*) [12,13]. After several months of the HIBP, they voluntarily end hibernation even when the surrounding environment remains in winter-like conditions (post-hibernation period: post-HIBP; figure 1*a*) [12,13]. Thus, once they experience several months of hibernation, Syrian hamsters develop refractoriness to hibernation. In other words, they lose their sensitivity to hibernation in response to continuous winter-like conditions, which is hereafter referred to as hibernation refractoriness. Gonadal photorefractoriness is another type of refractoriness. Syrian hamsters regress their gonads in response to the SP (less than 12.5 h/day) [14] but become insensitive to the regressive effects of the SP and undergo spontaneous recrudescence of the regressed gonads [15,16]. Under natural photoperiodic conditions, this spontaneous recrudescence of the gonads occurs long before the day length reaches 12.5 h/day, and most animals are fertile by the vernal equinox [17,18]. This photorefractory response, namely the spontaneous recrudescence of regressed gonads, is also observed in hamsters that do not hibernate under prolonged SP-cold conditions [19]; suggesting that hibernation is not necessary for gonadal photorefractoriness.

Mammals employ the circadian clock system to sense photoperiods and adapt their physiology to the seasonal changes [20]. Many living organisms exhibit an approximate 24 h rhythm in physiology and behaviour, driven by internal self-sustained oscillators termed the circadian clock [21]. Mammals adapt their rhythm (such as the sleep/wake cycle) and activity to the day-night cycle by entrainment using the circadian clock [22,23]. This entrainment is mediated by the suprachiasmatic nucleus (SCN), which is located in the hypothalamus and synchronizes peripheral clocks by converting retinal optical information into hormonal and neuronal signals [23,24]. The SCN governs the circadian rhythm of Tb [23,25–28], and the pattern of the diel Tb rhythm changes seasonally [29]. The diel Tb rhythm can, therefore, serve as a convenient indicator of the internal circadian clock and its changes [30]. In response to the seasonal change, mammals recognize the increase in day length and express summer physiology when the light coincides with a specific phase of high circadian photosensitivity, called the photoinducible phase (*φ*i) [20,31]. However, the gonadal photorefractoriness does not accompany a change in diel profiles of pineal melatonin or clock gene expression in the SCN and pars tuberalis regions of the pituitary gland [32,33].

The effects of hibernation on circadian systems remain unclear. Previous studies on ground squirrels and hamsters hibernating in natural or laboratory conditions proposed that the circadian rhythm disappears during the entire HIBP and HIB-DT [34–39]. By contrast, a few studies have reported the presence of circadian Tb rhythms during HIB-DT [40,41]. Many hibernating species remain in their nests where light cannot reach them during the HIBP. These species are, therefore, exposed to light-dark cycle after the HIBP. During HIB-DT, which constitutes most of the HIBP, the animals keep their eyes closed and curl up with their faces buried in their nests, suggesting that the retina receives no photic information. Therefore, even under laboratory conditions, the circadian system of animals during HIB-DT is not entrained to the light-dark cycle. By contrast, during HIB-PA, when animals are active and feed with their eyes open, transient entrainment of the circadian system to the light-dark cycle under laboratory conditions may occur. However, the duration of HIB-PA is usually less than one day [4], which is too short to achieve such entrainment. Therefore, it is likely that animals require re-entrainment of the circadian system to the light-dark cycle following hibernation refractoriness.

Syrian hamsters exhibit clear photoperiod-dependent changes in their diel rhythms. When hamsters are entrained to a long photoperiod (LP), the onset of activity and an increase in Tb are observed near the transition of the light to dark phase [42–47], which is designated the LP-type. By contrast, when hamsters are entrained to SP, activity onset is observed several hours after the transition, which is designated the SP-type [44–47]. These LP- and SP-type differential patterns of activity and Tb enabled us to evaluate whether the animals were entrained to the LP or SP at different time points: pre-HIBP, HIBP, and post-HIBP. In the wild, it is likely that Syrian hamsters disappear from the ground in late fall and reappear in late winter [48], suggesting that these hamsters may hibernate after SP entrainment and conclude hibernation in the nest when the day length increases. However, during hibernation in an underground nest, they are unable to sense the increasing ambient photoperiod in late winter. Thus, we hypothesized that Syrian hamsters have unknown endogenous mechanisms accompanying hibernation to meet the necessity for their rhythm to re-entrain to the ambient LP when they emerge from the nest following hibernation refractoriness. It is unclear how hamsters adapt to the seasonal photoperiodic changes experienced after HIBP. Considering that the diel Tb rhythm allows us to easily discriminate between the LP- and SP-types, we investigated how hibernation and its refractoriness affect the diel Tb rhythm. We accomplished this by using long-term high-resolution Tb records of Syrian hamsters that hibernated in constant and continuous winter-like SP-cold laboratory conditions.

## 2. Methods

### (a) Animals and housing in summer-like conditions

The hamsters were purchased from an outbred colony (Japan SLC., Inc Shizuoka, Japan) and housed under LP and warm (LP-warm) conditions (24–25°C with a 14 h light (L): 10 h dark (D) cycle (06 : 00–20 : 00), 100–650 lux) with ad libitum access to water and food (MR standard diet, Nihon Nosan, Kanagawa, Japan). Three to four individuals were housed per cage, and their body mass was recorded weekly when the cages were replaced.

### (b) Tb recording

Tb data loggers (iButton, #DS1925L-F5, Maxim Integrated, Chandler, AZ, USA) were coated with rubber (total mass approx. 3.5 g; Plasti Dip, Performix Coatings, Blaine, MN, USA) and surgically implanted into animals after 10–15 min of anaesthesia with inhalation of 3-4 % isoflurane. The Tb data loggers (iButtons) had a resolution of 0.0625°C, and the range (−10 to + 65°C) was calibrated by the manufacturer. According to the manufacturer's datasheet, the accuracy of its measurement is Tb ± 0.5°C. All the iButtons were programmed to record Tb every 10 min. The animals with iButton implants were housed individually in polypropylene cages to eliminate social interactions. They were allowed access to food and water ad libitum for one week in a summer-like condition and then relocated to a winter-like condition.

### (c) Hibernation induction in winter-like conditions

One to two weeks after the iButton implantation, the hamsters were transferred to winter-like conditions in an SP-cold room where the photoperiod and ambient temperature were maintained at 8L : 16D (lights on 10 : 00–18 : 00, 200−1100 lx) and 4°C. The body masses of the animals were measured in an SP-cold room and the cages were changed once a week or every other week, except for the animals in the HIB-DT group. To detect HIB-DT by visual inspection, we used the modified ‘saw-dust method’ as previously reported [12]. Cage replacement and body mass measurements of the animals in the HIB-DT group were omitted to avoid disturbing their hibernation. The Tb data were successfully recovered and analysed from 12 hibernating animals (five females and seven males) and seven animals that did not exhibit HIB-DT (five females and two males). Three hibernating hamsters were individually placed into a dark box (less than 0.01 lux) with an aeration path introduced during the later phases of the HIBP until post-HIBP. The Tb records of these animals were obtained from the iButtons, similar to those acquired for the other animals. To detect HIB-DT in a dark box in real time, the oxygen consumption rate was monitored using the respiratory gas analyser ARCO-2000 (ARCO System, Tokyo, Japan) [49] (electronic supplementary material, Supplemental method).

### (d) Terms and definitions of the hibernation-related study parameters

In this study, we defined hibernation based on the changes in Tb. When the Tb of the hamster was lower than 30°C, it was defined as a torpor bout [50]. A torpor bout with a Tb below 10°C for at least 10 consecutive hours was defined as HIB-DT. Torpor bouts other than HIB-DT were defined as shallow torpors. Hamsters that did not exhibit HIB-DT for more than seven months during the SP-cold conditions were classified as non-hibernating animals under prolonged SP-cold. The time at which the Tb first fell below 30°C, during the first HIB-DT induction, was defined as the onset of the HIBP. The time at which the Tb first exceeded 30°C, during arousal from the last HIB-DT, was defined as the end of the HIBP. The pre-HIBP was defined as the period from the start of the SP-cold condition to the onset of the HIBP. The HIBP was defined as the period from onset to the end of the HIBP. The post-HIBP was defined as the period from the end of the HIBP to the end of the SP-cold condition (figure 1*a*).

### (e) Detection of rhythmicity in Tb

The Tb data of the hibernating and non-hibernating animals under prolonged SP-cold were subjected to the *χ*-square periodogram procedure [51] to test the existence of diel rhythms (electronic supplementary material, Supplemental method).

### (f) Comparisons of the mean Tb between the light and dark phases

The Tb data were recorded on the days when cage replacement and body mass measurements were performed to eliminate their effects on Tb. A Tb of below 30°C was excluded from the data analysis in order to exclude Tb during torpor, in which animals are considered not to be entrained to the light–dark cycle. For hibernating and non-hibernating animals under prolonged SP-cold, the difference in the mean Tb between the light and dark phases was determined by subtracting the average Tb in the light phase from that of the dark phase (electronic supplementary material, figure S1 and Supplemental method).

### (g) Determination of the acrophases of the diel Tb rhythm

The acrophase of the diel Tb rhythm was determined using ClockLab Analysis (Actimetrics, Wilmette, IL, USA). The acrophases of the different periods (periods I–VIII) were compared using 3-day sections [52], with the clock times 0 : 00 to 24 : 00. The acrophases were compared based on zeitgeber time (ZT) with reference to light onset (ZT0).

I: The last 2 or 3 days before the SP-cold transfer (LP-warm conditions)
II: 2 weeks after the SP-cold transfer
III: 4 weeks after the SP-cold transfer
IV: 3 days before the onset of the HIBP
V: The first 3 days after the end of the HIBP
VI: 2 weeks after period V
VII: 4 weeks after period V
VIII: 6 weeks after period V

In approximately half of the animals, there was an overlap between the date when the body mass measurement was taken and period IV or V. In such cases, the Tb data were pre-processed as follows: if body mass measurements were performed during the above periods, the analysis period was shifted backward for period IV and forward for period V to a date when no overlap occurred. Periods I–VIII, during which shallow torpor occurred, were excluded from the analysis. To ensure the relevance of the acrophase analysis, the Tb data in periods I–VIII were submitted to the χ-square periodogram for periods between 0 h and 36 h to determine the presence of statistically significant rhythmicity [52]. Data not satisfying the conditions of 22 h < period1 < 26 h and amplitude1 > 2100 were excluded from further analysis. The phase difference (−12 ≤ hour < 12) in acrophases before and after the HIBP within an individual was calculated (electronic supplementary material, Supplemental method).

### (h) Phase angle (*ψ*) analysis

The onset of Tb was determined by using a modified version of a method described in previous studies [42,43], as follows. Four-hour moving averages of Tb for periods I, III, IV, V and VII were used to eliminate the influence of the ultradian rhythm [53]. For each period, three days (period I, 2 days for 7 individuals) of Tb data were divided into a single day by averaging the Tb values over the same time of day. The average Tb values for periods I, III, IV, V and VII were calculated as the mean Tb levels for each period. The crossing point of the diurnal Tb rise and mean Tb level was determined as the Tb onset. The time difference between the light offset and Tb onset was defined as the phase angle (*ψ*_onset_). The crossing point of the diurnal Tb fall and the mean Tb level was determined as the Tb end.

## 3. Results

### (a) The diel Tb rhythm is obscured during HIBP, even in euthermic HIB-PA

Long-term data acquired at high time and temperature resolutions enabled precise analysis of the Tb rhythm in the pre-HIBP, HIBP and post-HIBP (electronic supplementary material, figure S1*a*). Chi-square periodogram analysis was used to verify the existence of diel Tb rhythms during these periods. For animals that did not hibernate for more than seven months under SP-cold conditions, the period corresponding to the HIBP was analysed (electronic supplementary material, figure S1*b*). A 24 h rhythm was identified for all periods except the HIBP (electronic supplementary material, figure S1*c*–*f*), indicating that the diel Tb rhythm was specifically lost during the HIBP. Nocturnal Syrian hamsters exhibit a diel Tb rhythm entrained to the light–dark cycle, with a higher Tb during the active dark phase than Tb during the resting light phase [43,52,54]. The tendency for the average Tb to be higher in the dark phase than in the light phase was lost in HIB-PA (electronic supplementary material, figure S1*g*), whereas this tendency persisted throughout the experimental course in animals that did not hibernate under prolonged SP-cold conditions (electronic supplementary material, figure S1*h*). These results suggest that the diel Tb rhythm weakened or ceased specifically during the HIBP, even during euthermic HIB-PA, and recovered after the HIBP.

### (b) Acrophase analysis identified a hibernation-dependent cancellation of entrainment to the SP (phase shift) and subsequent re-entrainment of the diel Tb rhythm to the SP

To identify the characteristic trends in the diel Tb rhythm in hibernating animals, we calculated the acrophase of the diel Tb rhythm throughout the experimental period. A representative example is presented in figure 1*b*. After transferring the hamsters to a winter-like SP-cold condition from a summer-like LP-warm condition, the acrophase began to recede because of the time spent in the LP-warm condition and because of entrainment to the SP (closed square bracket A in figure 1*b*). Approximately 4 weeks after the SP-cold transfer, it finally settled to the second half of the dark phase (closed square bracket B in figure 1*b*). In the subsequent HIBP, the diel Tb rhythm was not detected (electronic supplementary material, figure S1*d*) and the calculated acrophases were incorrect. Immediately after the end of the HIBP (period V in figure 1*a*,*b*,*d*), the diel rhythmicity of the Tb recovered, and the calculated acrophases were located around the first half of the dark phase. Subsequently, the acrophases gradually receded (closed square bracket A’ in figure 1*b*) and settled to the second half of the dark phase (closed square bracket B’ in figure 1*b*; figure 1*d*). This shift in acrophases was similar to that observed after the transfer from LP-warm to SP-cold conditions (figure 1*b,d*). However, in the non-hibernating animals under prolonged SP-cold, the acrophase of the diel Tb rhythm became constant (closed square bracket B in figure 1*c*; figure 1*e*) after receding to the second half of the dark phase following the transfer to SP-cold conditions (closed square bracket A in figure 1*c*; figure 1*e*). These results suggest that the phase of the diel Tb rhythm changes during HIBP.

To quantify the observed shift, we analysed the acrophases of the diel Tb rhythm from 12 successfully hibernating individuals (seven females and five males). Polar plots at different periods with a 3-day section [52] of I–VIII (figure 1*f–m*) demonstrated that in the period I, acrophases were found around the ZT 18 (figure 1*f*). After being transferred to the winter-like, SP-cold conditions, the acrophase gradually receded and was fixed at approximately ZT 18 (figure 1*g–i*). This fixation was cancelled in period V—the period immediately after the end of the HIBP (figure 1*a*,*b*)—because the acrophase was detected around ZT 13–14 (figure 1*j*). The acrophases then gradually receded to around ZT 18 (figure 1*k–m*), as observed in hamsters that were entrained to the SP in the pre-HIBP (figure 1*g–i*).

To examine whether the acrophases immediately after the HIBP were shifted from those observed when hamsters were entrained to the SP, we compared the differences in acrophases between hamsters entrained to the SP before the HIBP (period III) and at different periods post-HIBP (periods V, VI, and VII) within the same individual (figure 1*n–p*). This comparison demonstrates that the acrophase immediately after the end of the HIBP tended to advance 4 h from the acrophase of hamsters entrained to the SP before the HIBP (figure 1*n*). In addition, the acrophases gradually receded and were re-fixed to the phase when the hamsters were entrained to the SP during the pre-HIBP (figure 1*o,p*). These results indicate that the entrainment of the diel Tb rhythm to the SP is transiently cancelled by hibernation and that every hamster resumes the Tb rhythm in a similar phase of the light–dark cycle immediately after the HIBP.

### (c) The phase of the diel Tb rhythm reverted to the phase observed in LP-warm conditions after HIBP

**Figure 2.**
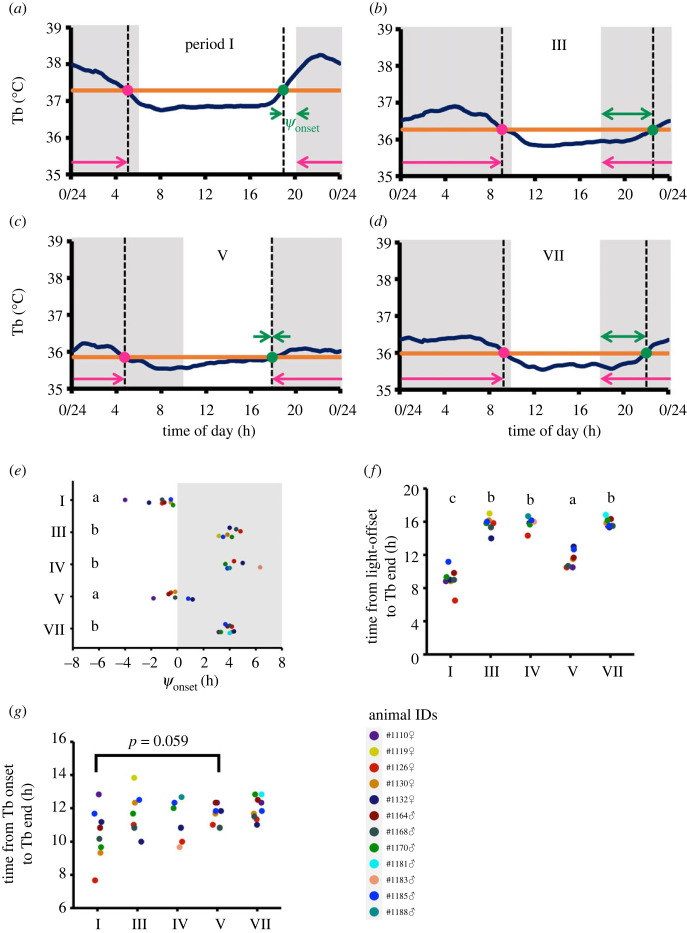
Phase reversal of diel Tb rhythm (PRT) identified by phase angle analysis. (*a*–*d*) A representative diel Tb rhythm of a hibernating hamster (#1168♂) in periods I, III, V and VII. The periods are the same as those shown in figure 1. The change in Tb educed by the 4-h moving average is shown by deep blue lines. In each period, the orange line and the green dot indicate the mean level of Tb and the Tb onset, respectively. The latter is the intersection of the diurnal rise of the educed Tb and the mean level of Tb. The phase angle (*ψ*_onset_) is the time difference between the light offset and Tb onset. The magenta dot indicates the Tb end, which is the intersection of the diurnal fall of the educed Tb and the mean level of Tb. The shaded areas in the diagrams indicate the dark phases. (*e*) Phase angles (*ψ*_onset_) in periods I, III–V and VII. The shaded areas in the diagrams indicate the dark phases. Combinations of different lowercase letters indicate combinations where significant differences were found in the ANOVA and Tukey-Kramer test (*p* < 0.05). (*f*) The time duration from light offset to Tb end in periods I, III–V and VII. Statistical analysis is done as in (*e*). (*g*) Time duration from Tb onset to Tb end between periods I, III–V and VII. The significance test between each period was performed using the Brunner-Munzel test. Period I: LP-warm conditions. III: Four weeks after the SP-cold transfer. IV: Immediately before the onset of the HIBP. V: Immediately after the end of the HIBP. VII: Four weeks after the completion of the HIBP.

Syrian hamsters exhibit activity onset close to the transition from the light phase to the dark phase (light offset) when entrained to the LP, whereas hamsters entrained to the SP exhibit activity onset several hours after the light offset [44–47]. As the onset of Tb rise (Tb onset) and activity onset concur [42], Tb onset could be an indicator of entrainment to the photoperiod. Tb onset was defined as the intersection of the diurnal Tb rise and the mean Tb level [42,43]. As expected, it was located around the light offset in the LP-warm condition (figure 2*a*,*e*) and approximately 4 h after the light offset in animals that were entrained to the SP but had not yet hibernated (a closed square bracket B and B’ in figure 1*b*; figure 2*b*,*d*,*e*). However, immediately after the end of the HIBP, Tb onset was observed around the light offset even though the animals were maintained under SP-cold conditions (figure 2*c*,*e*). In addition, the time duration from the light offset to the Tb end immediately after the end of the HIBP was closer to that observed in hamsters that were entrained to the LP as opposed to those entrained to the SP (figure 2*f*). By contrast, the time duration from Tb onset to Tb end was equivalent to that when hamsters were entrained to the SP and were slightly longer than that when hamsters were entrained to the LP (figure 2*g*). This indicates that the time for transition between the ascending and descending stages immediately after the end of the HIBP was not very different from that when hamsters were entrained to the LP or SP, but its phase reverted to the phase when hamsters were entrained to the LP. The spontaneous change in the diel Tb rhythm from the winter to summer phases after hibernation is hereafter termed as ‘phase reversal of the diel Tb rhythm (PRT).’

### (d) Phase reversal of the diel Tb rhythm occurs because of the phase convergence effect of the light–dark cycle on the circadian Tb rhythm that is restored by hibernation refractoriness

**Figure 3.**
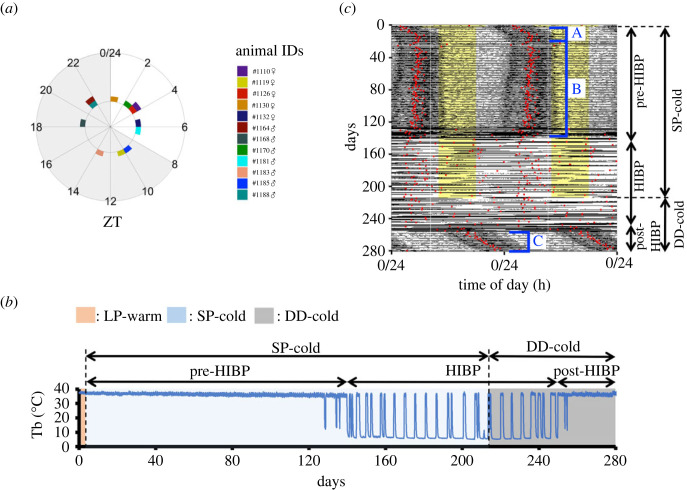
Light–dark cycle determines the phase of the diel Tb rhythm (PRT) irrespective of the last arousal time. (*a*) Distribution of ZT during the HIBP exit. The polar plot shows the ZT at which each hamster exited the HIBP, namely the ZT at which the Tb exceeded 30°C during the rewarming process in the last arousal from the last hypothermic deep torpor bout (HIB-DT). The shaded area indicates the dark phase. (*b,c*) Representative Tb changes of a hamster (#1535♀) that was subjected to constant-dark conditions since the middle of the HIBP (*b*) and its double-plot actogram in a 24 h time window (*c*). The black portion in each line of the actogram indicates the value of Tb, which is normalized to its maximum and minimum line values. In each line of the actogram, the zero and maximum values of the *y*-axis correspond to the minimum and maximum values of Tb within the line. The yellow shaded area indicates the light phase, and no shaded area corresponds to the dark phase. The red dots indicate the acrophases of the diel Tb rhythm fitted by a sine function at 24 h of τ. DD (dark and dark; 0L : 24D)-cold indicates the constant dark and cold conditions. The closed square brackets A, B, and C indicate: A) the period after the transition from LP-warm to SP-cold conditions, during which the acrophase of the diel rhythm gradually receded with entrainment to SP; B) the period after the SP entrainment during which the acrophases were fixed in the second half of the dark phase before the HIBP; and C) the period after the end of the HIBP under DD-cold conditions during which the free-running circadian Tb rhythm was observed.

We sought to identify the factors that contribute to PRT. First, we investigated whether the PRT depends on the phase of the light–dark cycle in which hamsters exit the last HIB-DT, namely the HIBP. ZT at arousal from the last HIB-DT varied among the individuals (figure 3*a*). This suggests that the PRT occurs independently of the phase of the light–dark cycle at which rewarming from the last HIB-DT occurs.

We then examined whether light or photoperiod was necessary for the PRT by exposing the hibernating hamsters to constant darkness, thereby eliminating the effects of light and photoperiod (figure 3*b*). The acrophases in constant darkness were different from those in LP-warm conditions immediately after the end of the HIBP (figure 3*c*), and the period of circadian Tb rhythm was 24.29 ± 0.06 h (mean ± s.d., *n* = 3). Thus, the animals exhibited a free-running pattern with no fixation at a specific time (closed square bracket C in figure 3*c*). These results suggest that light is not necessary for the re-establishment of the diel Tb rhythm itself after the HIBP but it determines the phases of the diel Tb rhythm immediately after the end of the HIBP for PRT. Thus, the circadian Tb rhythm is restored without photic stimuli after hibernation refractoriness, and the PRT is achieved by the effect of light–dark cycle that converges the Tb rhythm to a diel Tb rhythm, which is a similar to that experienced by hamsters when entrained to LP.

## 4. Discussion

In this study, we analysed high-resolution and long-term recording data of Tb in a facultative mammalian hibernator, the Syrian hamster, under SP-cold conditions. We found that the diel oscillation of the Tb rhythm observed during the pre-HIBP became undetectable in the HIBP and reappeared during the post-HIBP in hibernating hamsters (electronic supplementary material, figure S1*a–f*). This result is consistent with previous studies showing that the diel Tb rhythm disappears during and reappears after the HIBP in ground squirrels [34,36–39,55,56]. In addition, our high-resolution Tb data demonstrated that the difference in the mean Tb between the dark (active) and light (inactive) phases was lost during HIB-PA, a period during which hamsters could be active and feed with their eyes open (electronic supplementary material, figure S1*g*,*h*). Three possible but not necessarily mutually exclusive explanations for these observations are as follows. First, during HIB-PA, the circadian Tb rhythm responsible for generating the diel Tb rhythm disappears or attenuates to undetectable levels. Second, the diel Tb rhythm is disturbed or masked by locomotor activity during HIB-PA. During this period the animals actively move around immediately after arousal from HIB-DT, which largely affects the Tb. To clarify these points, future studies should simultaneously record the Tb and spontaneous activity of Syrian hamsters during the HIBP. Third, the duration of one HIB-PA bout may have been too short for the diel rhythm of the hamsters to be entrained to the light–dark cycles, resulting in a loss of difference in Tb between the light and dark phases. However, further studies are required to clarify this hypothesis.

The most surprising finding of this study is that the redevelopment of the diel Tb rhythm immediately after the HIBP accompanies the PRT from the SP-type (4 h delayed Tb onset from the light offset; figure 2*b,d*,*e*) to the LP-type (Tb onset near the light offset; figure 2*a*,*c*,*e*) [42,43]. PRT was confirmed by phase angle analysis based on the onset of Tb rise (Tb onset). Since Tb is positively correlated with activity [43,52] and Tb onset and activity onset concur [42], PRT may co-occur with the reversal of activity patterns. The PRT is considered to be a ‘phase shift’ because the phase of the rhythm is shifted to a specific phase (figure 1*j*,*n*, figure 2*e*) and because the duration from Tb onset to Tb end is relatively constant throughout the periods examined (figure 2*g*). However, PRT has a feature that is distinct from the simple phase shift that occurs in response to environmental changes, such as travelling to different time zones (jet lag) or seasonal photoperiodic changes, because the surrounding environment is still SP-cold when the HIBP ends in our setting. In addition, the LP-type diel Tb rhythm changed to the SP-type during the post-HIBP (figure 1*b*,*d*,*j–p,* figure 2*c–e*). These observations suggest that PRT induction is governed by an endogenous mechanism that is not affected by the ambient photoperiod. PRT has another feature distinct from a simple phase shift: its hibernation dependence, because non-hibernating animals under prolonged SP-cold did not show PRT (figure 1*c*,*e*). This hibernation dependence discriminates PRT from gonadal photorefractoriness that accompanies the regrowth of regressed gonads in both hibernating and non-hibernating animals under prolonged SP-cold [19]. In addition, it has been reported that the phase relationship between activity onset and light offset does not clearly change before and after gonadal photorefractoriness [45]. This evidence suggests that PRT constitutes hibernation refractoriness.

The mechanisms underlying PRT require further investigation. One important factor contributing to the PRT could be the rapid temperature changes during the HIB-DT and HIB-PA cycles. In cultured human cell lines or mouse SCN slices, rewarming from the cold to euthermic conditions resets the phase of circadian clock gene expression [57,58]. Similarly, a phase reset of the clock gene expression rhythm may occur during rewarming from the hypothermic conditions during hibernation. Melatonin in the pineal gland is maintained at low levels during HIB-DT and increases rapidly upon rewarming from HIB-DT, independent of the light–dark phase during which rewarming occurs [59]. Therefore, repeated elevation of melatonin levels during the last arousal period may contribute to the PRT. However, there is no plausible explanation for how the elevation of melatonin, as opposed to every arousal, triggers the PRT. At present, there are limited data supporting the involvement of melatonin in PRT.

Another crucial factor contributing to PRT may be the changes in responsiveness to the light–dark cycle. The hamsters spontaneously restored their circadian Tb rhythm without light exposure following the HIBP (figure 3*b*), suggesting that the light or light–dark cycles are not necessary for the recovery of the circadian Tb rhythm following hibernation refractoriness. This result is consistent with those acquired from Arctic ground squirrel observations. These animals restore their rhythmicity of the circadian Tb rhythm after hibernation refractoriness without light stimuli [39]. In Syrian hamsters, a light offset is a trigger for activity onset when entrained to the LP [47] and activity onset concurs with Tb onset [42]. However, the phase of the diel Tb rhythm was immediately aligned by light into the phase in the LP-warm condition under SP (figure 2*e*,*g*), whereas the ZT at which the HIBP ended varied between hamsters (figure 3*a*). Additionally, the phase of the circadian Tb rhythm varied in hamsters that terminated HIBP under constant dark conditions (figure 3*c*). Thus, immediately after the end of the HIBP, inter-individual synchronization of the diel Tb rhythm is achieved by the robust resetting effect of the light–dark cycle. Generally, the reception of a light pulse by the retina resets the phase of the diel rhythm based on a phase response curve [60]. In our experimental settings, light–dark cycles (8L : 16D) were maintained during the HIBP, allowing the animals to sense the light–dark cycle. However, it is currently unclear and logistically impossible to examine whether the phase response curves during and immediately after the end of the HIBP are identical to the phase response curves previously reported in hamsters during a non-HIBP. Unknown endogenous mechanisms governing the photoperiodic responses necessary for entrainment to the ambient photoperiod [31] may be changed during HIBP, contributing to PRT. Chronic administration of a serotonin agonist in Syrian hamsters results in an earlier onset of activity with respect to light offset [61]. Therefore, from a neurochemical aspect, it is notable to investigate whether the serotonin system is enhanced at the end of the HIBP under prolonged SP-cold conditions, and whether it shortens the time from light offset to activity onset, resulting in the close proximity of the Tb onset and light offset, as the hamsters are entrained to the LP.

In this study, we demonstrated that the PRT occurs in an hibernation-dependent manner in Syrian hamsters. Such a reversal is not observed in individuals that do not hibernate under prolonged winter-like conditions; it is, therefore, a phenomenon associated with hibernation. During the HIBP in the nest, access to the outer environmental photoperiod cues might be shut down in Syrian hamsters, similar to ground squirrels and chipmunks. However, these animals must adapt their physiology rapidly to the external environment after emergence. One possible solution is to pre-adapt to the external environment. Our data provide novel evidence that Syrian hamsters spontaneously remodel their photoperiodic Tb rhythm response from a winter-adapted SP-type to a summer-adapted LP-type in a hibernation-dependent manner. The PRT should result in rapid inter-individual synchronization of the diel Tb rhythm in a summer-like state immediately after the end of the HIBP. Considering that Syrian hamsters are vernal breeders that breed shortly after the end of the HIBP, and PRT was observed in both sexes (figure 2*e–g*), PRT may contribute to the expansion of reproductive opportunities during the breeding season. Thus, our findings support the hypothesis that mammalian hibernation consists of not only a simple energy conservation program in winter but also a program that anticipates the active season to come and facilitates its acclimation.

## Data Availability

We have uploaded the data as electronic supplementary material [62] along with our manuscript files.

## References

[1] Shinomiya A, Shimmura T, Nishiwaki-Ohkawa T, Yoshimura T. 2014 Regulation of seasonal reproduction by hypothalamic activation of thyroid hormone. Front. Endocrinol. **5**, 12. (10.3389/fendo.2014.00012) PMC3930870 24600435

[2] Geiser F. 2004 Metabolic rate and body temperature reduction during hibernation and daily torpor. Annu. Rev. Physiol. **66**, 239-274. (10.1146/annurev.physiol.66.032102.115105) 14977403

[3] Heldmaier G, Ortmann S, Elvert R. 2004 Natural hypometabolism during hibernation and daily torpor in mammals. Respir. Physiol. Neurobiol. **141**, 317-329. (10.1016/j.resp.2004.03.014) 15288602

[4] Ruf T, Geiser F. 2015 Daily torpor and hibernation in birds and mammals. Biol. Rev. Camb. Philos. Soc. **90**, 891-926. (10.1111/brv.12137) 25123049 PMC4351926

[5] Prendergast BJ, Nelson RJ, Zucker I. 2002 Mammalian seasonal rhythms: behavior and neuroendocrine substrates. Hormones Brain Behav. **2**, 93-156. (10.1016/B978-008088783-8.00014-0)

[6] Pengelley ET, Fisher KC. 1963 The effect of temperature and photoperiod on the yearly hibernating behavior of captive golden-mantled ground squirrels (*Citellus Lateralis Tescorum*). Can. J. Zool. **41**, 1103-1120. (10.1139/z63-087)

[7] Pengelley ET, Asmundson SJ, Barnes B, Aloia RC. 1976 Relationship of light intensity and photoperiod to circannual rhythmicity in the hibernating ground squirrel, *Citellus lateralis*. Comp. Biochem. Physiol. A Comp. Physiol. **53**, 273-277. (10.1016/s0300-9629(76)80035-7) 2435

[8] Kortner G, Geiser F. 2000 The temporal organization of daily torpor and hibernation: circadian and circannual rhythms. Chronobiol. Int. **17**, 103-128. (10.1081/cbi-100101036) 10757457

[9] Kondo N, Sekijima T, Kondo J, Takamatsu N, Tohya K, Ohtsu T. 2006 Circannual control of hibernation by HP complex in the brain. Cell **125**, 161-172. (10.1016/j.cell.2006.03.017) 16615897

[10] Schwartz C, Andrews MT. 2013 Circannual transitions in gene expression: lessons from seasonal adaptations. Curr. Top. Dev. Biol. **105**, 247-273. (10.1016/B978-0-12-396968-2.00009-9) 23962845 PMC4130376

[11] Wood SH et al. 2015 Binary switching of calendar cells in the pituitary defines the phase of the circannual cycle in mammals. Curr. Biol. **25**, 2651-2662. (10.1016/j.cub.2015.09.014) 26412130 PMC4612467

[12] Janský L, Haddad G, Kahlerová Z, Nedoma J. 1984 Effect of external factors on hibernation of golden hamsters. J. Comp. Physiol. B **154**, 427-433. (10.1007/bf00684450)

[13] Staples JF. 2016 Metabolic flexibility: hibernation, torpor, and estivation. Comp. Physiol. **6**, 737-771. (10.1002/cphy.c140064) 27065167

[14] Gaston S, Menaker M. 1967 Photoperiodic control of hamster testis. Science **158**, 925-928. (10.1126/science.158.3803.925) 6054164

[15] Reiter RJ. 1972 Evidence for refractoriness of the pituitary-gonadal axis to the pineal gland in golden hamsters and its possible implications in annual reproductive rhythms. Anat. Rec. **173**, 365-371. (10.1002/ar.1091730311) 5039088

[16] Stetson MH, Matt KS, Watson-Whitmyre M. 1976 Photoperiodism and reproduction in golden hamsters: circadian organization and the termination of photorefractoriness. Biol. Reprod. **14**, 531-537. (10.1095/biolreprod14.5.531) 1276317

[17] Reiter RJ. 1973 Pineal control of a seasonal reproductive rhythm in male golden hamsters exposed to natural daylight and temperature. Endocrinology **92**, 423-430. (10.1210/endo-92-2-423) 4682859

[18] Reiter RJ. 1974 Influence of pinealectomy on the breeding capability of hamsters maintained under natural photoperiodic and temperature conditions. Neuroendocrinology **13**, 366-370. (10.1159/000122222) 4841981

[19] Larkin JE, Jones J, Zucker I. 2002 Temperature dependence of gonadal regression in Syrian hamsters exposed to short day lengths. Am. J. Physiol. Regul. Integr. Comp. Physiol. **282**, R744-R752. (10.1152/ajpregu.00299.2001) 11832395

[20] Wood SH et al. 2020 Circadian clock mechanism driving mammalian photoperiodism. Nat. Commun. **11**, 4291. (10.1038/s41467-020-18061-z) 32855407 PMC7453030

[21] Patke A, Young MW, Axelrod S. 2020 Molecular mechanisms and physiological importance of circadian rhythms. Nat. Rev. Mol. Cell Biol. **21**, 67-84. (10.1038/s41580-019-0179-2) 31768006

[22] Harmer SL, Panda S, Kay SA. 2001 Molecular bases of circadian rhythms. Annu. Rev. Cell Dev. Biol. **17**, 215-253. (10.1146/annurev.cellbio.17.1.215) 11687489

[23] Lu Q, Kim JY. 2022 Mammalian circadian networks mediated by the suprachiasmatic nucleus. FEBS J. **289**, 6589-6604. (10.1111/febs.16233) 34657394

[24] Hastings MH, Maywood ES, Brancaccio M. 2018 Generation of circadian rhythms in the suprachiasmatic nucleus. Nat. Rev. Neurosci. **19**, 453-469. (10.1038/s41583-018-0026-z) 29934559

[25] Ruby NF, Dark J, Burns DE, Heller HC, Zucker I. 2002 The suprachiasmatic nucleus is essential for circadian body temperature rhythms in hibernating ground squirrels. J. Neurosci. **22**, 357-364. (10.1523/JNEUROSCI.22-01-00357.2002) 11756519 PMC6757609

[26] Scheer FA, Pirovano C, Van Someren EJ, Buijs RM. 2005 Environmental light and suprachiasmatic nucleus interact in the regulation of body temperature. Neuroscience **132**, 465-477. (10.1016/j.neuroscience.2004.12.012) 15802197

[27] Refinetti R, Kaufman CM, Menaker M. 1994 Complete suprachiasmatic lesions eliminate circadian rhythmicity of body temperature and locomotor activity in golden hamsters. J. Comp. Physiol. A **175**, 223-232. (10.1007/BF00215118) 8071897

[28] Refinetti R. 1995 Effects of suprachiasmatic lesions on temperature regulation in the golden hamster. Brain Res. Bull. **36**, 81-84. (10.1016/0361-9230(94)00168-z) 7882054

[29] Wollnik F, Schmidt B. 1995 Seasonal and daily rhythms of body temperature in the European hamster (*Cricetus cricetus*) under semi-natural conditions. J. Comp. Physiol. B **165**, 171-182. (10.1007/BF00260808) 7665733

[30] Refinetti R. 2020 Circadian rhythmicity of body temperature and metabolism. Temperature **7**, 321-362. (10.1080/23328940.2020.1743605) PMC7678948 33251281

[31] Elliott JA. 1981 Circadian rhythms, entrainment and photoperiodism in the Syrian hamster. In Biological clocks in seasonal reproductive cycles (eds BK Follett, DE Follett), pp. 203-217. Bristol, UK: Wright.

[32] Carr AJ, Johnston JD, Semikhodskii AG, Nolan T, Cagampang FR, Stirland JA, Loudon AS. 2003 Photoperiod differentially regulates circadian oscillators in central and peripheral tissues of the Syrian hamster. Curr. Biol. **13**, 1543-1548. (10.1016/s0960-9822(03)00619-5) 12956958

[33] Lincoln GA, Johnston JD, Andersson H, Wagner G, Hazlerigg DG. 2005 Photorefractoriness in mammals: dissociating a seasonal timer from the circadian-based photoperiod response. Endocrinology **146**, 3782-3790. (10.1210/en.2005-0132) 15919753

[34] Hut RA, Barnes BM, Daan S. 2002 Body temperature patterns before, during, and after semi-natural hibernation in the European ground squirrel. J. Comp. Physiol. B **172**, 47-58. (10.1007/s003600100226) 11824403

[35] Revel FG, Herwig A, Garidou ML, Dardente H, Menet JS, Masson-Pevet M, Simonneaux V, Saboureau M, Pevet P. 2007 The circadian clock stops ticking during deep hibernation in the European hamster. Proc Natl Acad Sci U S A **104**, 13 816-13 820. (10.1073/pnas.0704699104) PMC1959465 17715068

[36] Kart Gur M, Refinetti R, Gur H. 2009 Daily rhythmicity and hibernation in the Anatolian ground squirrel under natural and laboratory conditions. J. Comp. Physiol. B **179**, 155-164. (10.1007/s00360-008-0298-0) 18797881

[37] Williams CT, Barnes BM, Buck CL. 2012 Daily body temperature rhythms persist under the midnight sun but are absent during hibernation in free-living arctic ground squirrels. Biol. Lett. **8**, 31-34. (10.1098/rsbl.2011.0435) 21752811 PMC3259947

[38] Williams CT, Barnes BM, Richter M, Buck CL. 2012 Hibernation and circadian rhythms of body temperature in free-living Arctic ground squirrels. Physiol. Biochem. Zool. **85**, 397-404. (10.1086/666509) 22705489

[39] Williams CT, Radonich M, Barnes BM, Buck CL. 2017 Seasonal loss and resumption of circadian rhythms in hibernating arctic ground squirrels. J. Comp. Physiol. B **187**, 693-703. (10.1007/s00360-017-1069-6) 28332018

[40] Menaker M. 1959 Endogenous rhythms of body temperature in hibernating bats. Nature **184**, 1251-1252. (10.1038/1841251a0)

[41] Grahn DA, Miller JD, Houng VS, Heller HC. 1994 Persistence of circadian rhythmicity in hibernating ground squirrels. Am. J. Physiol. **266**, R1251-R1258. (10.1152/ajpregu.1994.266.4.R1251) 8184969

[42] Decoursey PJ, Pius S, Sandlin C, Wethey D, Schull J. 1998 Relationship of circadian temperature and activity rhythms in two rodent species. Physiol. Behav. **65**, 457-463. (10.1016/s0031-9384(98)00187-5) 9877411

[43] Refinetti R. 1999 Relationship between the daily rhythms of locomotor activity and body temperature in eight mammalian species. Am. J. Physiol. **277**, R1493-R1500. (10.1152/ajpregu.1999.277.5.R1493) 10564224

[44] Daan S, Aschoff J. 1975 Circadian rhythms of locomotor activity in captive birds and mammals: their variations with season and latitude. Oecologia **18**, 269-316. (10.1007/BF00345851) 28308918

[45] Ellis GB, Turek FW. 1979 Changes in locomotor activity associated with the photoperiodic response of the testes in male golden hamsters. J. Comp. Physiol. **132**, 277-284. (10.1007/bf00614498)

[46] Scarbrough K, Losee-Olson S, Wallen EP, Turek FW. 1997 Aging and photoperiod affect entrainment and quantitative aspects of locomotor behavior in Syrian hamsters. Am. J. Physiol. **272**, R1219-R1225. (10.1152/ajpregu.1997.272.4.R1219) 9140023

[47] Elliott JA. 1976 Circadian rhythms and photoperiodic time measurement in mammals. Fed. Proc. **35**, 2339-2346. 964387

[48] Gattermann R, Fritzsche P, Neumann K, Al-Hussein I, Kayser A, Abiad M, Yakti R. 2001 Notes on the current distribution and the ecology of wild golden hamsters (*Mesocricetus auratus*). J. Zool. **254**, 359-365. (10.1017/s0952836901000851)

[49] Sunagawa GA, Takahashi M. 2016 Hypometabolism during daily torpor in mice is dominated by reduction in the sensitivity of the thermoregulatory system. Sci. Rep. **6**, 37011. (10.1038/srep37011) 27845399 PMC5109469

[50] Wassmer T, Wollnik F. 1997 Timing of torpor bouts during hibernation in European hamsters (*Cricetus cricetus* L.). J. Comp. Physiol. B **167**, 270-279. (10.1007/s003600050074) 9203368

[51] Sokolove PG, Bushell WN. 1978 The chi square periodogram: its utility for analysis of circadian rhythms. J. Theor. Biol. **72**, 131-160. (10.1016/0022-5193(78)90022-x) 566361

[52] Refinetti R. 1994 Contribution of locomotor activity to the generation of the daily rhythm of body temperature in golden hamsters. Physiol. Behav. **56**, 829-831. (10.1016/0031-9384(94)90251-8) 7800756

[53] Refinetti R. 2003 Metabolic heat production, heat loss and the circadian rhythm of body temperature in the rat. Exp. Physiol. **88**, 423-429. (10.1113/eph8802521) 12719767

[54] Chayama Y, Ando L, Tamura Y, Miura M, Yamaguchi Y. 2016 Decreases in body temperature and body mass constitute pre-hibernation remodelling in the Syrian golden hamster, a facultative mammalian hibernator. R. Soc. Open Sci. **3**, 160002. (10.1098/rsos.160002) 27152216 PMC4852639

[55] Hut RA, Van der Zee EA, Jansen K, Gerkema MP, Daan S. 2002 Gradual reappearance of post-hibernation circadian rhythmicity correlates with numbers of vasopressin-containing neurons in the suprachiasmatic nuclei of European ground squirrels. J. Comp. Physiol. B **172**, 59-70. (10.1007/s003600100227) 11828985

[56] Healy JE, Burdett KA, Buck CL, Florant GL. 2012 Sex differences in torpor patterns during natural hibernation in golden-mantled ground squirrels (*Callospermophilus lateralis*). J. Mammal. **93**, 751-758. (10.1644/11-mamm-a-120.1)

[57] Fischl H, McManus D, Oldenkamp R, Schermelleh L, Mellor J, Jagannath A, Furger A. 2020 Cold-induced chromatin compaction and nuclear retention of clock mRNAs resets the circadian rhythm. EMBO J. **39**, e105604. (10.15252/embj.2020105604) 33034091 PMC7667876

[58] Enoki R, Kon N, Shimizu K, Kobayashi K, Yamaguchi Y, Tomomi N. 2022 Cold-induced suspension and resetting of Ca^2+^ and transcriptional rhythms in the suprachiasmatic nucleus neurons. *bioRxiv*. (10.1101/2022.09.18.508357)

[59] Vaněček J, Janský L, Illnerová H, Hoffmann K. 1984 Pineal melatonin in hibernating and aroused golden hamsters (*Mesocricetus auratus*). Comp. Biochem. Physiol. A Comp. Physiol. **77**, 759-762. (10.1016/0300-9629(84)90197-x) 6143647

[60] Pittendrigh CS, Daan S. 1976 A functional analysis of circadian pacemakers in nocturnal rodents. II. The variability of phase response curves. J. Comp. Physiol. **106**, 253-266. (10.1007/bf01417857)

[61] Vijaya Shankara J, Orr A, Mychasiuk R, Antle MC. 2017 Chronic BMY7378 treatment alters behavioral circadian rhythms. Eur. J. Neurosci. **46**, 2782-2790. (10.1111/ejn.13744) 29044737

[62] Nakagawa S, Yamaguchi Y. 2023 Spontaneous recurrence of a summer-like diel rhythm in the body temperature of the Syrian hamster after hibernation. Figshare. (10.6084/m9.figshare.c.6858273) PMC10581774 37848068

